# Framing substance use disorders among forcibly displaced people through a syndemics lens.

**DOI:** 10.1192/j.eurpsy.2023.63

**Published:** 2023-07-19

**Authors:** J. Tay Wee Teck

**Affiliations:** School of Medicine, University of St Andrews, St Andrews, United Kingdom

## Abstract

**Abstract:**

Syndemics are synergistically interacting epidemics (for example, the epidemics of substance use disorders and forced displacement) in a particular context with shared drivers such as pre-existing political, structural, social and health conditions. Policymakers may ask what the risks of and needs are for forcibly displaced people with regards to substance use disorder (SUD). Working from a syndemics framework, we would argue that multiple risk and resiliency factors relating to both forced displacement and SUD work synergistically, and impact more significantly upon some populations than others. These risk factors include structural inequality and racism, social deprivation, violence, homelessness, trauma, childhood adversity, and co-morbid physical and mental health disorders. Individuals may become vulnerable while experiencing displacement, during transition, settlement or resettlement, and also through their life across and across generations. In this perspective, our answers to policymakers becomes more nuanced. We may argue for integrated, multi-modal interventions, cross-disciplinary collaborations, cross-pollination of ideas and knowledge and embedding lived experience to bridge gaps and make use of limited resources in sustainable ways. This presentation will detail aspects of the application of a syndemic lens to the evidence base on SUD among forcibly displaced people and generate discussion on what scientists, clinicians and policymakers can and may do with these insights.

**Disclosure of Interest:**

None Declared

